# Hypertrophic pyloric stenosis in infants: is it a congenital or acquired disorder? Reflections on 2 cases

**DOI:** 10.1186/2193-1801-3-555

**Published:** 2014-09-24

**Authors:** Faustin Félicien Mouafo Tambo, Irène Nadine Kouna Tsala, Marcelin Ngowe Ngowe, Gervais Andze Ondobo, Maurice Aurelien Sosso

**Affiliations:** Obstetrics, Gynaecology and Paediatric Hospital, Yaoundé, Cameroon

**Keywords:** Hypertrophic pyloric stenosis, Etiology, Sténose hypertrophique du pylore, Etiologie

## Abstract

Based on evidence from two collected and treated clinical observations of hypertrophic pyloric stenosis in children of 5 and 12 months of age, the authors give their point of view on the unresolved issue of the etiology of hypertrophic pyloric stenosis. They emphasize that there are more and more factors to prove this is an acquired condition.

## Résumé

À partir de 2 observations cliniques colligées et traitées de sténose hypertrophique du pylore chez les nourrissons de 5 mois et 12 mois, les auteurs font part de leurs réflexions sur la question non encore résolue de l’étiologie de la sténose hypertrophique du pylore. Ils soulignent l’existence de plus en plus de facteurs en faveur d’une affection acquise.

## Introduction

Hypertrophic pyloric stenosis (HPS) is defined as an hyperplasia of smooth muscle fibres of the pylorus that is responsible for the narrowing of the pyloric canal and obstruction of gastric emptying (Roquelaure and Sarles 2000; Li 2008). Its incidence is estimated at 1.5-3 per 1000 live births and the age of discovery is typically between the 3rd and 4th week of life (Mahalik et al. 2010; Schwartz 2006). This condition is known to be rare in Africa, and the cases of late discovery are even rarer in this context because of the early involvement of life prognosis, in the absence of resuscitation. Thus, the late observation of 2 cases of hypertrophic pyloric stenosis in infants of 5 months and 1 year in the paediatric surgery department of the Obstetrics, Gynaecology and Paediatric Hospital of Yaoundé (HGOPY) has prompted us to reopen the debate on the etiology of hypertrophic pyloric stenosis which is still mysterious, quite known. The aim of this study was to emphasize that there is more and more evidence that prove that this is an acquired condition whose mechanism is different from that of a developmental abnormality.

### Observation N° 1

K.J, a 5-month old male infant and first born in a family of one was admitted on 28.09. 06 for early non bilious vomiting that had been lasting for 5 days. Upon interrogation, it appeared there had been no ingestion of any caustic products, no gastro oesophageal reflux disease, and no previous case of hypertrophic pyloric stenosis was reported in the family. Physical examination performed on this infant whose general state was preserved, who had stained conjunctiva, was afebrile at 37° and weighed 5 Kgs led to the discovery of peristaltic waves spontaneously moving from left to right and, most importantly, of a firm and moving oblong mass under the right costal margin which was strongly suggestive of a pyloric olive. Abdominal sonography made after those clinical observations highlighted a hypertrophic pylorus with a 5.7 mm-thick wall associated with gastric stasis. Therefore, surgery indication was made after a complete pre surgery assessment, a short hydro electrolytic rebalancing through a periumbilical approach and, an extra mucosal Fredet-Ramstedt pyloromyotomy was carried on 12.10.11 with no complications followed by a resumption of feeding at H12. Five years later, no vomiting has been reported, the weight curve of the child is normal and he tolerates a regular diet.

### Observation N° 2

M.K., a 1-year old female infant was admitted in November 2008 for non bilious projectile vomiting that had been lasting for two weeks. The anamnesis did not reveal any abdominal distension, weight loss or previous medication. The medical history Chapter was not contributory. The clinical examination performed on a dehydrated infant revealed an epigastric arch. The pyloric olive was not palpated. Abdominal sonography in this context revealed a 25 mm-long and 10 mm-thick pyloric olive. After resuscitation, the surgical indication was made and an extra mucosal Fredet-Ramstedt pyloromyotomy was carried out by periumbilical route. The immediate post-surgery period was uneventful and there was a resumption of feeding at H10. Three years later, no vomiting has been reported and M.K. has a normal weight gain.

## Discussion

The condition described in 1887 by Hirschsprung has been subjected to many pathogenic hypotheses, none of which has been validated to date. Following the analysis of the two presented clinical observations, the genetic theory seems unlikely to explain the occurrence of hypertrophic pyloric stenosis because no family case of hypertrophic pyloric stenosis was reported in both cases. That the patient in Observation No. 2 is a female is contrary to the literature data (Le Dosseur et al. 2009) which posit that the disease is 3–4 times more common in boys than in girls. Conversely the first born in a family of one in Observation N°1 seems to have a multi gene predisposition, one of the major genes being linked to chromosome X, and is in the prevalence of the disease in firstborn boys although that may be due to chance, given the small size of our sample. The environmental theory that tends to connect the occurrence of HPS to stressful events during pregnancy has not been established in our two cases. The neurogenic theory that maintains that pyloric hypertrophy is the consequence of an antropyloric dyssynergia which itself is a consequence of the degeneration of the innervation did not explain convincingly the occurrence of HPS in our 2 cases. As a matter of fact, this theory cannot explain the absence of obstructive events in degenerative diseases like Chagas disease; another of its shortcomings is the fact that no animal testing could reproduce the disease by creating a barrier or hormonal destruction (Le Dosseur et al. 2009). Late diagnosis, a common practice in our under medicalised countries, is an argument in favor of a congenital origin of the condition in two observed cases. Indeed, the operator dependent nature of ultrasound, its unavailability in emergency units and the inadequacy of technical facilities in most developing countries combine with the low socio-economic status of populations to explain the delay in the diagnosis of the condition in the two reported cases. But more than anything else, it is the incomplete and functional stenosis of the pyloric canal which justifies, in our opinion, the late occurrence in both reported cases. The unicist theory that relates infant HPS to stenosing pyloric hypertrophy in adults was hard to prove valid in the absence of esophageal pH monitoring and upper gastro intestinal endoscopy (UGIE) to formally eliminate the possible association between HPS, gastro oesophageal reflux desease (GERD), peptic esophagitis and ulcer. Similarly, hormonal theory that tends to explain the occurrence of HPS by the hypergastrinemia in the newborn has not been verified in our work, due to the lack of laboratory equipped for such an assay.

According to (Mahalik et al. 2010), hypertrophic pyloric stenosis is an acquired condition. Indeed, pyloric stenosis is characterized by a hypertrophy of circular fibers that would result in the narrowing and obstruction of the pyloric canal due to the compression of the longitudinal fibers of the mucosa. This acquired origin of HPS had been identified and discussed since 2000 by Paulozzi (Paulozzi 2000) who argued that HPS result from a Helicobacter pylori infection.

That same year, (Hoey 2000) showed that the risk of HPS is increased by a factor 8 for children who were administered erythromycin between 3–13 days after birth. The other antibiotics showed no risks of HPS. Similarly, in 2002, (Sorensen et al. 2002) showed over a period of 10 years that the risk of the HPS was multiplied by 2 for infants whose mother is a smoker.

## Conclusion

No theory appears to explain all alone the Hypertrophic Pyloric and its quite stereotypical period of occurrence. Its etiology is probably multifactorial as the pylorus seems sensitive to attack at certain times and in certain subjects.
